# Zusammensetzung der Immunzellen in der Haut und dem subkutanen Fettgewebe von Patienten mit systemischer Sklerose

**DOI:** 10.1111/ddg.15864_g

**Published:** 2026-04-08

**Authors:** Marija Geroldinger‐Simić, Ana E. Aguilar González, Gerald Exler, Georg Stary

**Affiliations:** ^1^ Abteilung für Dermatologie und Venerologie Ordensklinikum Linz Elisabethinen Linz Österreich; ^2^ Medizinische Fakultät Johannes‐Kepler‐Universität Linz Österreich; ^3^ Universitätsklinik für Dermatologie Medizinische Universität Wien Wien Österreich; ^4^ CeMM‐Forschungszentrum für Molekulare Medizin der Österreichische Akademie der Wissenschaften Wien Österreich; ^5^ Christian Doppler Labor für Chronisch‐entzündliche Hautkrankheiten Wien Österreich

**Keywords:** Haut, spektrale Durchflusszytometrie, subkutanes Fettgewebe, systemische Sklerose, High‐content spectral flow cytometry, skin, subcutaneous adipose tissue, systemic sclerosis

## Abstract

**Hintergrund und Ziele:**

Systemische Sklerose (SSc) ist eine seltene, chronische Autoimmunerkrankung, die durch Fibrose der Haut und/oder der inneren Organe gekennzeichnet ist. Neue Erkenntnisse deuten darauf hin, dass subkutanes Fettgewebe zur systemischen Entzündung und Fibrose bei SSc beitragen kann. Ziel dieser Studie war eine umfassende Analyse der Zusammensetzung von Immunzellen bei SSc durch gleichzeitige Untersuchung von Blut, Haut und subkutanem Fettgewebe.

**Patienten und Methoden:**

Mittels spektraler Durchflusszytometrie erstellten wir Profile der wichtigsten Untergruppen von Immunzellen und untersuchten deren Assoziationen mit klinischen Merkmalen von SSc.

**Ergebnisse:**

Patienten mit leichter Hautfibrose (niedrige mRSS) wiesen vermehrt cDC1‐, moDC‐ und ThGM‐CSF‐Zellen in der Haut auf, zusammen mit einem Zustrom von Th22‐Zellen und reduzierten terminalen NK‐Zellen im subkutanen Fettgewebe. SSc‐Patienten mit Lungenfibrose zeichneten sich durch weniger NK‐Zellen und mehr CD8^+^ T‐Zellen im peripheren Blut aus. Während wir in anti‐Scl‐70‐positiven Patienten vermehrt CD8^+^ Effektor‐T‐Zellen in der Haut fanden, wiesen anti‐Zentromer‐positive Patienten erhöhte ThGM‐CSF‐Zellen in der Haut auf.

**Schlussfolgerung:**

Diese Erkenntnisse unterstreichen die potenzielle Rolle unterschiedlicher Immunzellsubtypen für die Krankheitsprogression und die gewebespezifische Fibrose bei SSc.

## EINLEITUNG

Die systemische Sklerose (SSc) ist eine systemische, chronische Autoimmunerkrankung, die durch eine fortschreitende Fibrose der Haut und/oder der inneren Organe, vor allem der Lunge und des Magen‐Darm‐Trakts, gekennzeichnet ist. Die SSc wird je nach Ausmaß der Hautbeteiligung in zwei Hauptgruppen unterteilt: limitierte kutane SSc (lcSSc) und diffuse kutane SSc (dcSSc).^1^ Während beide Subtypen gemeinsame pathophysiologische Merkmale aufweisen, ist die dcSSc eine progrediente Erkrankung und weist ein höheres Risiko für die Beteiligung innerer Organe, einschließlich schwerer Lungenfibrose, auf.^2^ Die zugrundeliegende Pathogenese der SSc ist multifaktoriell und umfasst eine Dysregulation des Immunsystems, Gefäßanomalien und eine übermäßige Ablagerung von extrazellulärer Matrix, die alle zu der bei der Krankheit beobachteten fibrotischen Progression beitragen.^3^, ^4^

Das Immunsystem spielt eine zentrale Rolle in der Pathogenese von SSc, wobei sowohl angeborene als auch adaptive Immunkomponenten zur Gewebeschädigung und Fibrose beitragen.^3^ Die frühen Krankheitsstadien sind durch Autoimmunität gekennzeichnet, wobei Autoantikörper wie Anti‐Topoisomerase I (Anti‐Scl‐70), Anti‐Zentromer‐Antikörper (CENP) und/oder Anti‐RNA‐Polymerase‐III‐Antikörper gebildet werden, die eng mit bestimmten klinischen Phänotypen und dem Fortschreiten der Krankheit verbunden sind.^5^ Studien zur Erstellung von Immunprofilen bei SSc haben sich weitgehend auf Veränderungen im Blut konzentriert.^6^ Gewebespezifische Immunveränderungen in der Haut und im subkutanen Fettgewebe sind größtenteils unbekannt. Neuere Studien deuten darauf hin, dass das subkutanes Fettgewebe eine entscheidende immunregulatorische Rolle spielt.^7^ Eine Dysfunktion der im Fettgewebe ansässigen Immunzellen könnte zur systemischen Entzündung und Fibrose bei SSc beitragen.^8^

Ziel dieser Studie ist eine umfassende Analyse der Zusammensetzung von Immunzellen in Blut, Haut und subkutanem Fettgewebe von SSc‐Patienten. Mittels spektraler Durchflusszytometrie erstellten wir Profile wichtiger Immunzellsubtypen, und untersuchten Assoziationen der Immunzellen mit klinischen Parametern von SSc. Wir stellten die Hypothese auf, dass eine Dysregulation der Immunzellsignatur in der Haut und im subkutanen Fettgewebe bei SSc‐Patienten eine Schlüsselrolle bei der Fibrose der Haut spielt, während systemische Immunveränderungen mit dem Fortschreiten der Krankheit und der Lungenfibrose in Verbindung stehen. Durch diese Profile von Immunzellen mit klinischen Krankheitsparametern wollen wir neue Erkenntnisse über Immunmechanismen gewinnen, die für die Entstehung der Fibrose bei SSc verantwortlich sind.

## PATIENTEN UND METHODIK

### Patientenkohorte und Rekrutierung

Patienten mit SSc und gesunde Kontrollpersonen wurden an der Abteilung für Dermatologie des Ordensklinikums Elisabethinen Linz, Österreich, rekrutiert. Alle Patienten und gesunden Kontrollpersonen nahmen freiwillig an dieser Studie teil und gaben ihre schriftliche Einwilligung nach vollständiger Aufklärung. Diese Studie wurde von der Ethikkommission der Johannes‐Kepler‐Universität Linz, Österreich, genehmigt (Protokoll 1108/2021 und Änderungen). Die Diagnose von SSc wurde gemäß den 2013 vom *American College of Rheumatology* (ACR) und der *European League Against Rheumatism* (EULAR) aufgestellten Klassifikationskriterien gestellt. Die Einschlusskriterien für Patienten mit SSc waren: Alter 18–90 Jahre; bestätigte Diagnose von SSc. Jeder Patient wurde hinsichtlich klinischer Aspekte charakterisiert (Anamnese, klinischer Status einschließlich modifiziertem Rodnan Skin Score [mRSS], Labortests einschließlich Autoantikörper). Ausschlusskriterien für die Kontrollgruppe waren: SSc, metabolisches Syndrom, Diabetes mellitus Typ II, akute Infektionen.

Die klinische Routinediagnostik umfasste die Untersuchung auf eine Reihe klinischer Antikörper, wie antinukleäre Antikörper (ANA) und ANA‐Untergruppen (Anti‐Zentromer‐, Anti‐Scl70‐, Anti‐Rnp/Sm‐, Anti‐Rnp70‐, Anti‐SSA/Ro, Anti‐SSB/La‐ und Anti‐Sm‐Antikörper), unter Verwendung standardisierter ELISA‐ und Immunfluoreszenztests. Die Lungenfibrose (LF) wurde durch hochauflösende Computertomographie (HRCT) und Lungenfunktionstests bewertet, während die pulmonale arterielle Hypertonie (PAH) mithilfe von Stressechokardiographie und Rechtsherzkatheteruntersuchung beurteilt wurde.

### Haut‐ und Fettbiopsate und Probenlagerung

Gewebeproben (tiefe Hautbiopsate einschließlich Fettgewebe) wurden Patienten mit SSc unter örtlicher Betäubung mit einer 6‐mm‐Stanze entnommen (ein Routineverfahren in der dermatologischen Ambulanz). Es wurden zwei Hautproben entnommen, eine von betroffener und eine von nicht betroffener Haut. Als Biopsiestellen wurden die Innenseite des Oberarms oder des Bauches (günstige Fettverteilung, geringe Wundspannung) bevorzugt. Die Gewebeproben der Kontrollgruppe stammten aus Gewebematerial, das bei routinemäßigen Hautoperationen (Vollhauttransplantationen, Narbenkorrekturen oder Lappenoperationen) entnommen wurde, und die meisten von ihnen waren hinsichtlich der Biopsiestelle identisch. Für die Zwecke der Studie wurden aus diesem überschüssigen Material für die Kontrollgruppe Hautproben einschließlich des subkutanen weißen Fettgewebes entnommen.

Das subkutane Fettgewebe wurde mit einem Skalpell von der Dermis getrennt. Die Biopsate wurden gewogen, anschließend in fötalem Rinderserum (FBS, Gibco, Kat.‐Nr. 10500064) mit 10% Dimethylsulfoxid (DMSO, Sigma, Kat.‐Nr. 317275‐500ML) eingefroren und bis zur Analyse bei −80 °C gelagert.

### Isolierung von Immunzellen aus PBMC

Vollblut wurde mit BD™ Vacutainer™ Glas‐Zellpräparationsröhrchen (BD, Kat.‐Nr. 362782) entnommen, und die periphere mononukleäre Blutzellen (PBMC) wurde durch Gradientenzentrifugation gemäß dem Herstellerprotokoll isoliert. Die isolierte PBMC wurde in FBS + 10% DMSO eingefroren und bis zur Analyse bei −80 °C gelagert.

### Probenvorbereitung für die Durchflusszytometrie

Die Hautbiopsate wurden bei 37 °C aufgetaut und zweimal mit phosphatgepufferter Kochsalzlösung (PBS; Gibco, Kat.‐Nr. 14190144) gewaschen. Anschließend wurden die Biopsate gewogen, mit einem Skalpell fein zerkleinert und mit dem *Whole Skin Dissociation Kit*, human (Miltenyi Biotec, Kat.‐Nr. 130‐101‐540) ohne Enzym P verdaut, um die Oberflächenepitope für die Durchflusszytometrie zu erhalten. Die subkutanen Fettbiopsate wurden ebenfalls bei 37 °C aufgetaut, zweimal mit PBS gewaschen, gewogen und anschließend weiterverarbeitet. Das Fett wurde in einem 2‐ml‐Röhrchen verdaut, das Kollagenase IV (10 U/ml; Fisher Scientific, Kat.‐Nr. LS004189) und Desoxyribonuklease I (10 mg/ml; Sigma Aldrich, Kat.‐Nr. DN25‐1G) in RPMI 1640 (Gibco, Kat.‐Nr. 52400‐025), ergänzt mit 5% FBS, enthielt. Beide Gewebebiopsate wurden über Nacht bei 37 °C unter leichtem Schütteln verdaut.

Anschließend wurden die Zellen durch einen 100‐µm‐Filter filtriert. Die Hautsuspension wurde dreimal mit jeweils 10 mL eiskaltem PBS, ergänzt mit 10% FBS, gewaschen, während die Fettsuspension dreimal mit jeweils 10 mL eiskaltem PBS, ergänzt mit 2% FBS, gewaschen wurde. Die Hautzellen wurden danach 5 Minuten bei 300 g und 4 °C zentrifugiert, und das Pellet wurde für die Antikörperfärbung in PBS resuspendiert.

Die Fettsuspension wurde 10 Minuten bei 800 g und 4 °C zentrifugiert, um schwimmende Adipozyten zu entfernen. Der Überstand wurde verworfen, und die verbleibenden Zellen wurden in 30 mL eiskaltem PBS + 2% FBS resuspendiert und erneut 10 Minuten bei 800 g und 4 °C zentrifugiert, um eine reine Fraktion stromaler Gefäßzellen zu isolieren. Das resultierende Pellet wurde anschließend für die Antikörperfärbung in PBS resuspendiert.

Periphere mononukleäre Blutzellen (PBMC) wurden bei 37 °C in 5 mL RPMI 1640 aufgetaut und 5 Minuten bei 300 g zentrifugiert. Danach wurden die Zellen einmal mit PBS gewaschen und für die Antikörperfärbung in kaltem PBS resuspendiert.

### Probenvorbereitung für die Durchflusszytometrie

Die Hautbiopsate wurden bei 37°C aufgetaut und zweimal mit phosphatgepufferter Kochsalzlösung (PBS, Gibco, Kat: 14190144) gewaschen. Die Biopsate wurden dann gewogen und anschließend mit einem Skalpell fein zerkleinert und mit dem *Whole Skin Dissociation Kit*, human (Miltenyi Biotec, Kat: 130‐101‐540) ohne Enzym P verdaut, um die Oberflächenepitope für die Durchflusszytometrie zu erhalten. Die subkutanen Fettbiopsate wurden bei 37°C aufgetaut und vor dem Wiegen und der weiteren Verarbeitung zweimal mit PBS gewaschen. Das Fett wurde in einem 2‐mL‐Röhrchen verdaut, das Kollagenase IV (10U/mL, Fisher Scientific, Kat.: LS004189) und Desoxyribonuklease I (10mg/mL, Sigma Aldrich, Kat.: DN25‐1G) in RPMI 1640 (Gibco, Kat.: 52400‐025), ergänzt mit 5% FBS, enthielt. Beide Gewebebiopsate wurden über Nacht bei 37°C unter leichtem Schütteln verdaut.

Danach wurden die Zellen durch einen 100 µm‐Filter filtriert. Die Hautsuspension wurde mit 3x10 mL eiskaltem PBS, ergänzt mit 10% FBS, gewaschen, während die Fettsuspension mit 3x10 mL eiskaltem PBS, ergänzt mit 2% FBS, gewaschen wurde. Die Hautzellen wurden dann 5 Minuten lang bei 300 g und 4 °C zentrifugiert, und das Pellet wurde anschließend für die Antikörperfärbung in PBS resuspendiert. Die Fettzellsuspension wurde 10 Minuten lang bei 800 g und 4 °C zentrifugiert, um schwimmende Adipozyten zu entfernen. Der Überstand wurde verworfen und die Zellen wurden erneut in eiskaltem 30 mL PBS + 2% FBS resuspendiert und erneut 10 Minuten bei 800 g bei 4 °C zentrifugiert, um eine reine Fraktion stromaler Gefäßzellen zu isolieren. Das Pellet mit dieser Fraktion wurde dann für die Antikörperfärbung in PBS resuspendiert.

Die peripheren mononukleären Blutzellen wurden bei 37 °C in 5 mL RPMI 1640 aufgetaut und 5 Minuten bei 300 g zentrifugiert. Anschließend wurden die Zellen einmal mit PBS gewaschen und für die Antikörperfärbung in kaltem PBS resuspendiert.

### Durchflusszytometrie

Die Zellen wurden 30 Minuten lang bei 4 °C im Dunkeln mit einem Antikörpermix gefärbt, der einen Lebendfarbstoff enthielt, um die Lebensfähigkeit der Zellen zu beurteilen (Tabelle S1 für Antikörper und Verdünnungen). Die Zellen wurden dann mit PBS gewaschen, das 2 mM EDTA (ThermoFisher, Kat: 15575020) und 1% Rinderserumalbumin (BSA, Sigma, A2153) enthielt, und in PBS mit 3 mM EDTA resuspendiert. Die Proben wurden dann direkt mit dem Spektralanalysator Aurora (Cytek^®^) mit einem 5‐Laser‐Setup (355 nm, 405 nm, 488 nm, 561 nm und 640 nm) aufgenommen. Die Oberflächenfärbung erfolgte nach Standardprotokollen mit einer Reihe von fluorochromkonjugierten Antikörpern. Alle Antikörper wurden für eine optimale Verdünnung pro Gewebe titriert. Um eine genaue spektrale Entmischung zu gewährleisten, wurden für jedes Fluorochrom einfach gefärbte Referenzkontrollen sowie Autofluoreszenzkontrollen (AF) für ungefärbte Zellen erfasst.

Das Durchflusszytometrie‐Panel wurde für Gewebe und Blut getrennt optimiert, soweit die Zellzahlen dies zuließen, und FMO‐Färbungen (Fluoreszenz minus eins) wurden für jedes Gewebe durchgeführt, um die Färbespezifität zu bestimmen.

### Statistische Analyse

Die Durchflusszytometriedaten wurden in FlowJo (Version 10.8.1) und GraphPad Prism (Version 10.0.3) analysiert. Die statistische Signifikanz wurde mittels Two‐Way ANOVA mit Tukey‐Korrektur für multiple Hypothesentests bewertet. Die Normalitätsprüfung wurde mit dem Shapiro‐Wilk‐Test in GraphPad Prism durchgeführt. Die zweifache ANOVA wurde gewählt, da die Patientengruppen und die Untergruppen der Immunzellen in derselben Tabelle verglichen wurden und die zweifache ANOVA einen Mehrfachvergleich über Zeilen hinweg ermöglicht.

## ERGEBNISSE

### Patientenkollektiv

Die Studiengruppe umfasste 13 Patienten mit SSc und 13 Kontrollpersonen ohne SSc. Das Durchschnittsalter der SSc‐Patienten betrug 59 Jahre (Bereich 48–79 Jahre), und neun der 13 SSc‐Patienten waren weiblich. Sechs SSc‐Patienten hatten begrenzte kutane SSc (lcSSc) und sieben SSc‐Patienten hatten diffuse kutane SSc (dcSSc). Die mediane Krankheitsdauer der SSc‐Patienten betrug 6 Jahre (Bereich 2–38). ANA‐Antikörper waren bei zwölf von 13 SSc‐Patienten positiv, fünf SSc‐Patienten waren positiv für Anti‐Scl70‐Antikörper und vier SSc‐Patienten zeigten eine Positivität für CENP. Die Merkmale der Patienten mit SSc und der Kontrollgruppe (ohne SSc) sind in Tabelle 1 zusammengefasst.

**TABELLE 1:** Übersicht über die Patientenkohorte.

Studiengruppe	SSc Patienten	lcSSc	dcSSc	Kontrollen
Anzahl, N	13	6	7	13
Weiblich/ Männlich, N	9/ 4	5/ 1	4/ 3	6/ 7
Alter, Medianwert (Spanne)	59 (48‐79)	64 (55‐79)	56 (48‐67)	70 (62‐88)
Limitierte SSc/ Diffuse SSc, N	6/ 7			
Krankheitsdauer, Medianwert (Spanne)	6 (3‐38)	7 (3‐24)	6 (3‐16)	
ANA positiv, N	12	6	6	
Anti‐Scl70/ CENP, N	5/ 4	2/ 2	3/ 2	
mRSScore (0‐51), Medianwert (Spanne)	8 (4‐44)	5 (4‐11)	16 (7‐44)	
Klinische Manifestationen				
Calcinosis cutis (CC), N	6	3	3	
Digitale Ulzera (DU), N	3	0	3	
Dysphagie, N	12	6	6	
Lungenfibrose (LF), N	6	2	4	
PAH, N	3	1	2	
Raynaud, N	11	5	6	
Reflux, N	7	4	3	
Sicca, N	7	3	4	
Mycophenolat mofetil, N	4	1	3	
Bosentan, N	4	1	3	
Macitentan, N	3	0	3	
Methotrexat, N	6	2	4	
Nintedanib, N	1	0	1	
Prednisolon, N	3	1	2	
Sildenafil, N	3	0	3	

### Veränderungen in der Zusammensetzung der Immunzellen bei SSc‐Patienten

**ABBILDUNG 1:**
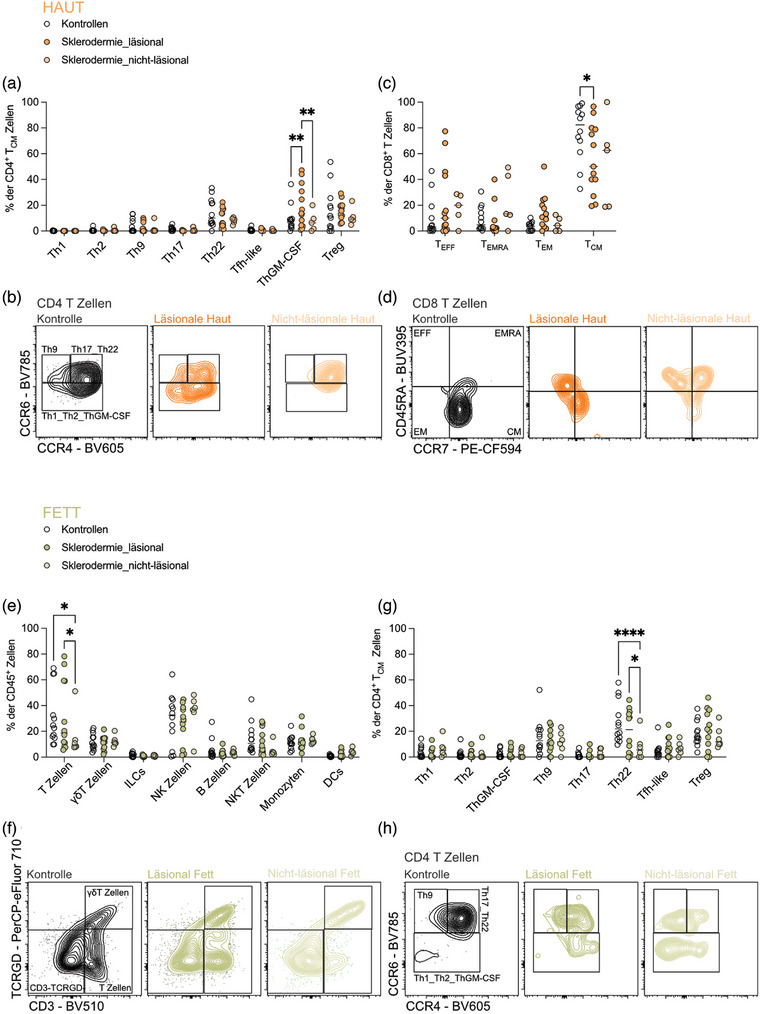
Veränderungen der Zusammensetzung von Immunzellen in der Haut und im subkutanen Fettgewebe von Patienten mit systemischer Sklerose (SSc).

Biopsate der läsionalen und nicht‐läsionalen Haut sowie des angrenzenden subkutanen Fettgewebes wurden zusammen mit einer Blutprobe von SSc‐Patienten und gesunden Kontrollpersonen entnommen, um die Zusammensetzung der Immunzellen zu analysieren. Aus den Blutproben wurden PBMC isoliert, und die Gewebeproben wurden verdaut. Anschließend wurden alle Proben mittels spektraler Durchflusszytometrie unter Verwendung eines 32‐Farben‐Panels analysiert, das wichtigen Untergruppen von Immunzellen in Blut, Haut und Fett abdeckt (Abbildung S1a–d, Tabelle S1, Tabelle S2). Um systemische und gewebespezifische Immunveränderungen der SSc besser zu verstehen, untersuchten wir zunächst die Zusammensetzung der Immunzellen aus dem Blut zwischen SSc‐Patienten und gesunden Kontrollpersonen. Wir beobachteten eine Zunahme der CD4^+^ T‐Zellen bei SSc‐Patienten, während NK‐Zellen insgesamt abnahmen (Abbildung S2a). Innerhalb der NK‐Zellpopulation waren reife NK‐Zellen bei SSc‐Patienten im Vergleich zu Kontrollpersonen relativ stark angereichert (Abbildung S2b). Während die Blutanalyse Einblicke in die systemische Immunaktivierung lieferte, dehnten wir unsere Untersuchung auf die Haut und das angrenzende subkutane Fettgewebe aus, von dem bekannt ist, dass es immunregulatorische Funktionen hat und dass im Fett ansässige Immunzellen während einer Entzündung dysfunktional sein können.^9^ In der läsionalen Haut von SSc‐Patienten fanden wir einen signifikanten Anstieg der proinflammatorischen Helfer‐T‐Zellen der ThGM‐CSF‐Untergruppe (Abbildung 1a,b) und eine Verringerung der CD8^+^ zentralen Gedächtnis‐T‐Zellen (Abbildung 1c,d). Interessanterweise zeigten sich die Unterschiede in der Zusammensetzung der Immunzellen im subkutanen Fettgewebe vor allem in den nicht‐läsionalen Arealen im Vergleich zu gesunden Kontrollen. Diese Bereiche wiesen eine allgemeine Abnahme der T‐Zellpopulationen im Fettgewebe auf (Abbildung 1e,f), mit einem deutlichen Rückgang der Th22‐Zellen (Abbildung 1g,h). Da Th22‐Zellen bekanntermaßen eine entscheidende Rolle bei der Wundheilung in der Haut spielen,^10^ kann deren Reduktion im nicht‐läsionalen Fettgewebe diese Gewebestellen für Entzündungen, Fibrose und Ulzerationen prädisponieren, was die Krankheitspathologie möglicherweise verschlimmert.

### Leichte Hautfibrose ist mit einer Zunahme entzündungsfördernder Zellen in der Haut verbunden

**ABBILDUNG 2:**
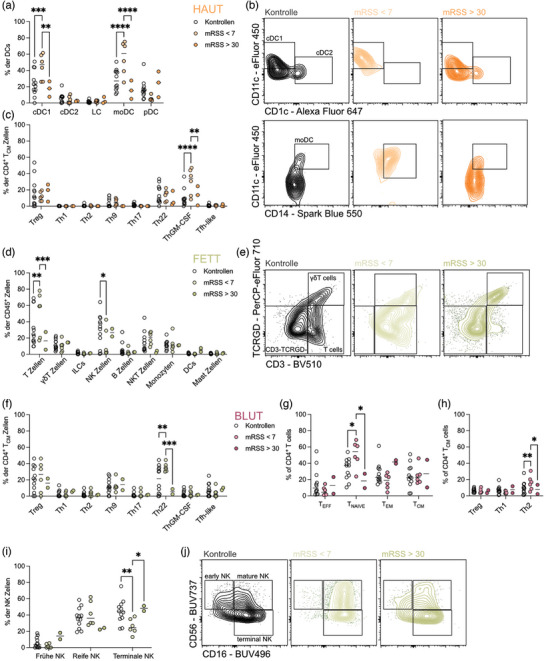
Eine milde Hautfibrose ist mit einem Einstrom proinflammatorischer Zellen in die Haut assoziiert.

Die minimalen Veränderungen, die beim Vergleich von SSc‐Patienten mit gesunden Kontrollpersonen im Immunkompartiment beobachtet wurden, spiegeln wahrscheinlich die Heterogenität der Krankheit und der Patientenkohorte wider. Um aussagekräftigere Veränderungen des Immunsystems zu ermitteln, gruppierten wir die Patienten nach klinischen Parametern und konzentrierten uns dabei auf den modifizierten Rodnan‐Hautscore (mRSS), ein Standardmaß für die Hautverdickung, das mit Fibrose und Krankheitsaktivität bei SSc korreliert.^11^ In unserer Kohorte wiesen Patienten mit niedrigem mRSS (<  7) im Vergleich zu den Kontrollen einen signifikanten Anstieg der konventionellen dendritischen Zellen vom Typ 1 (cDC1) und der aus Monozyten stammenden DC (moDC) in der Haut auf (Abbildung 2a,b). cDC1 und moDC gelten als Vermittler systemischer Entzündungen, indem sie Th1‐ und Th17‐Reaktionen auslösen.^12^, ^13^, ^14^, ^15^, ^16^ Dementsprechend fanden wir auch einen Anstieg von ThGM‐CSF‐Zellen (Abbildung 2c) in der Haut, die neben anderen entzündungsfördernden Funktionen die Differenzierung und Aktivierung myeloischer Zellen unterstützen.^17^, ^18^ Bei SSc‐Patienten mit leichter Hautfibrose (erkennbar an einem niedrigen mRSS) könnten diese Zellen adaptive, entzündungsfördernde Zellen rekrutieren und so die Krankheit vorantreiben. Dies spiegelt sich im subkutanen Fettgewebe wider, wo ein Zustrom von T‐Zellen und insbesondere Th22‐Zellen festgestellt wurde (Abbildung 2d–f). Im Blut beobachteten wir einen Anstieg der naiven CD4^+^ T‐Zellen (Abbildung 2g) und der Th2‐Zellen (Abbildung 2h). Außerdem fanden wir eine Abnahme der terminalen NK‐Zellen im subkutanen Fettgewebe von Patienten mit niedrigem mRSS (Abbildung 2i,j). Terminale NK‐Zellen haben nachweislich eine antifibrotische Funktion,^19^ daher könnte der Verlust dieser Zellen zur Auslösung einer systemischen proinflammatorischen Reaktion beitragen, wie sie bei SSc zu beobachten ist.

Zusammengenommen deuten diese Ergebnisse darauf hin, dass eine leichte Hautfibrose, die sich in einem niedrigen mRSS widerspiegelt, durch eine verstärkte Rekrutierung und Aktivierung von Entzündungszellen gekennzeichnet ist, die durch cDC1 und moDC in der Haut vermittelt werden. Dieser Prozess könnte zusätzliche Immunzellen aus dem Fettgewebe und dem Blutkreislauf anziehen und so die Voraussetzungen für das Fortschreiten der Fibrose schaffen.

### Eine Lungenbeteiligung bei SSc spiegelt sich hauptsächlich in Veränderungen der Immunzellen im peripheren Blut wider

**ABBILDUNG 3:**
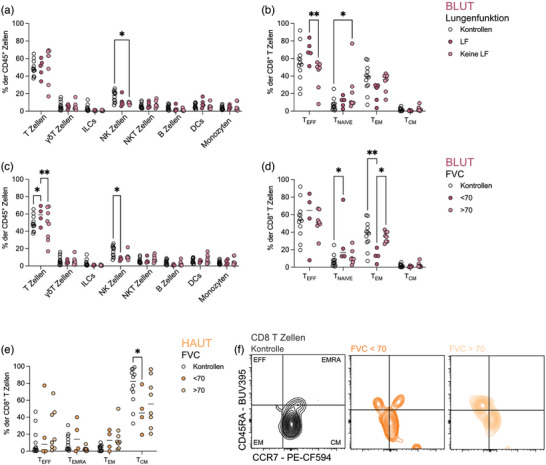
Die Lungenbeteiligung bei systemischer Sklerose spiegelt sich hauptsächlich in Veränderungen der Immunzellzusammensetzung im peripheren Blut wider.

Während bei Patienten mit leichter Hautfibrose (niedriger mRSS) Immunveränderungen vor allem in der Haut und im subkutanen Fettgewebe zu beobachten waren, wiesen Patienten mit Lungenfibrose (LF) die meisten Veränderungen im Blut auf. Patienten mit LF hatten einen systemischen Rückgang der NK‐Zellen im peripheren Blut (Abbildung 3a), aber einen Anstieg der CD8^+^ Effektor‐T‐Zellen im Vergleich zu Patienten ohne LF (Abbildung 3b), was verdeutlicht, wie die späteren Krankheitsstadien durch Veränderungen im adaptiven Arm des Immunsystems bestimmt werden. Dazu passend zeigten Patienten mit verminderter Lungenfunktion (forcierte Vitalkapazität, FVC < 70) ebenfalls einen Anstieg der T‐Zellen im Blut (Abbildung 3c). In dieser Gruppe sind insbesondere CD8^+^ naive T‐Zellen vermehrt, was auf Kosten der CD8^+^ Effektor‐Gedächtniszellen geht, die im Blut von Patienten mit einer FVC < 70 abgenommen haben (Abbildung 3d). In der Haut zeigen Patienten mit eingeschränkter Lungenfunktion einen Rückgang der CD8^+^ zentralen Gedächtnis‐T‐Zellen (Abbildung 3e,f).

Diese Ergebnisse unterstreichen die Rolle von NK‐Zellen und CD8^+^ T‐Zellen, die bei fortgeschrittener Krankheit mit Lungenbeteiligung im Blut zu finden sind, was darauf hindeutet, dass die adaptive Immunität bei der Vermittlung der Fibrose mit fortschreitender SSc zunehmend an Bedeutung gewinnt.

### SSc‐spezifische Autoantikörper stehen in unterschiedlichem Zusammenhang mit der Zusammensetzung der Immunzellen in der Haut

**ABBILDUNG 4:**
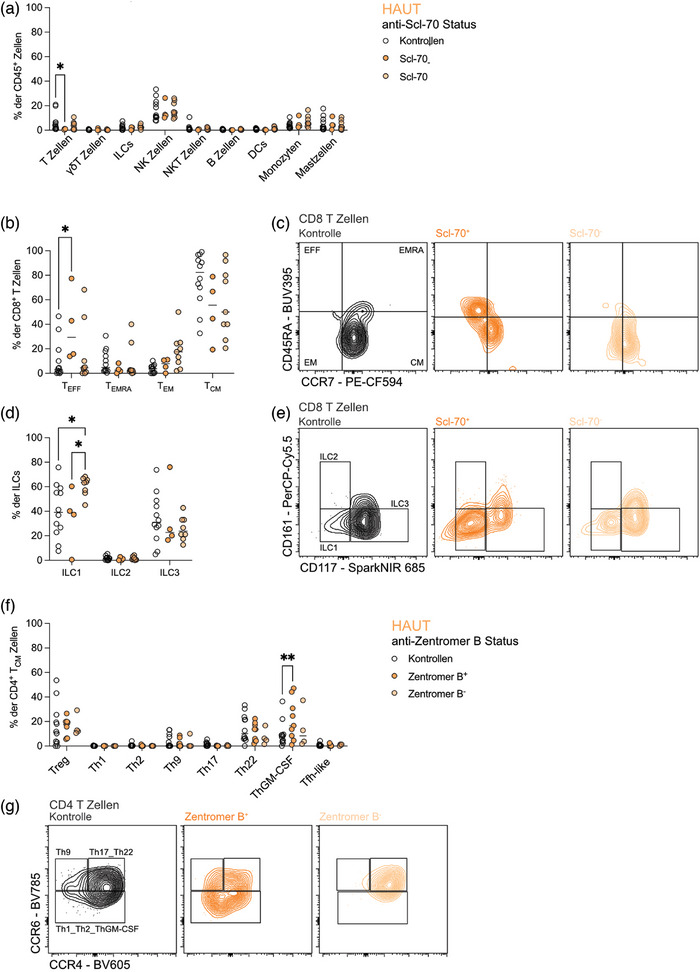
SSc‐spezifische Autoantikörper sind unterschiedlich mit der Zusammensetzung von Immunzellen in der Haut assoziiert.

Eines der wichtigsten immunologischen Merkmale bei SSc ist das Vorhandensein spezifischer Autoantikörper wie Anti‐Scl‐70, CENP und/oder Anti‐RNA‐Polymerase III.^5^ Patienten, die positiv auf Anti‐Scl‐70‐Antikörper reagierten, zeigten einen globalen Verlust an T‐Zellen (Abbildung 4a), aber einen Anstieg der CD8^+^ Effektor‐T‐Zellen in der Haut im Vergleich zu gesunden Kontrollen (Abbildung 4b,c). Im Vergleich zu Patienten mit negativem Anti‐Scl‐70‐Test wiesen diese Patienten auch einen Rückgang von ILC1 in der Haut auf (Abbildung 4d,e). Andererseits hatten CENP‐positive Patienten einen signifikanten Anstieg der ThGM‐CSF‐Zellen in der Haut (Abbildung 4f,g), was mit der Vorstellung übereinstimmt, dass diese Patienten häufig an lcSSc leiden.^5^ Dies deutet darauf hin, dass ThGM‐CSF‐Zellen mit einem milderen Verlauf der SSc‐Krankheit zusammenhängen. Die unterschiedlichen Immunprofile in der Haut von SSc‐Patienten auf der Grundlage des Autoantikörperstatus unterstreichen die Heterogenität der Krankheit.

## DISKUSSION

Diese Studie liefert eine detaillierte Analyse der Immunzellzusammensetzung in Blut, läsionaler und nichtläsionaler Haut sowie im subkutanen Fettgewebe von Patienten mit SSc. Obwohl uns die geringe Stichprobengröße bewusst ist, konnten dennoch signifikante Unterschiede festgestellt werden, die mit klinischen Parametern übereinstimmen. Wir weisen auf signifikante Veränderungen der Immunzelluntergruppen in Abhängigkeit von verschiedenen klinischen Parametern hin, die mit den Stadien des Krankheitsverlaufs assoziiert sind. Die Ergebnisse unterstreichen die Komplexität der Dysregulation von Immunzellen bei SSc und ihre potenziellen Auswirkungen auf das Verständnis des Krankheitsverlaufs, der Gewebefibrose und der Rolle spezifischer Immunzellsubgruppen bei der Modulation der Krankheitsaktivität.

Eine der wichtigsten Beobachtungen in dieser Studie ist die veränderte Zusammensetzung der Immunzellen bei Patienten mit leichter Hautfibrose (niedrige mRSS), die durch eine systemische Immunaktivierung und lokalisierte Veränderungen in der Haut und im subkutanen Fettgewebe gekennzeichnet ist. Die Zunahme von cDC1 und moDC in der Haut dieser Patienten ist bemerkenswert, da beide Zelltypen dafür bekannt sind, dass sie eine entscheidende Rolle bei der Aktivierung von Th1‐ und Th17‐Zellen spielen,^12^, ^13^, ^14^, ^15^, ^16^ welche die Entzündungsreaktionen bei Autoimmunkrankheiten maßgeblich beeinflussen. Eine frühere Studie zeigte, dass moDCs eine T‐Zell‐Polarisierung in Richtung Th2 und Th17 induzieren, insbesondere in den frühen Stadien der dcSSc.^20^ Die frühe Infiltration von proinflammatorischen ThGM‐CSF‐Zellen in der Haut unterstützt die Idee, dass diese Zellen zur Aktivierung von myeloischen Zellen und zur Rekrutierung von Entzündungszellen aus dem Blut beitragen und dadurch die lokale Gewebeentzündung verschlimmern. Außerdem hat sich gezeigt, dass GM‐CSF die Fibrose bei SSc stimuliert.^21^, ^22^ Dementsprechend fanden wir auch einen Verlust an Th22‐Zellen im nichtläsionalen subkutanen Fettgewebe, was für die Auslösung einer Entzündungsreaktion in lokalen Geweben spricht. Th22‐Zellen sind für ihre entzündungshemmenden Eigenschaften bekannt,^10^ und ihre Verminderung kann einen entzündungsfördernden Zustand im Fettgewebe begünstigen, der die Haut und das Fett für eine weitere Fibroseentwicklung vorbereiten könnte. Dies deckt sich mit früheren Studien, die darauf hindeuten, dass Th22‐Zellen eine Rolle bei der Regulierung der Entzündung des Fettgewebes und der metabolischen Dysfunktion spielen.^23^, ^24^, ^25^ Im Gegensatz dazu fanden wir bei Patienten mit niedrigem mRSS eine Zunahme von Th22‐Zellen im subkutanen Fettgewebe. IL‐22 kann die Fibroblastenreaktion auf TNF verstärken und so möglicherweise Hautfibrose fördern.^26^, ^27^ Da unsere Ergebnisse zu den Untergruppen der T‐Helferzellen jedoch auf der Expression von Oberflächenmarkern beruhen, müssen sie mit Vorsicht betrachtet und in künftigen Studien durch Auswertung der Zytokinproduktion validiert werden. In Übereinstimmung mit Ergebnissen der Zusammensetzung der Helfer‐T Zellen fanden wir auch eine Abnahme der terminalen NK‐Zellen im subkutanen Fettgewebe von SSc‐Patienten mit niedrigem mRSS. Terminalen NK‐Zellen wird eine antifibrotische Rolle zugeschrieben,^19^ weswegen ihr Verlust im Frühstadium der SSc das Auftreten von Entzündungen und Fibrose sowohl in der Haut als auch im Fettgewebe begünstigen könnte, was zur Pathogenese der Krankheit beiträgt.

Bei Patienten mit LF oder reduzierter FVC wurden die Immunveränderungen vorwiegend im Blut festgestellt, was den systemischen Charakter des Krankheitsverlaufs unterstreicht. Die Verringerung der NK‐Zellen im peripheren Blut von Patienten mit LF deckt sich mit früheren Befunden einer NK‐Dysfunktion bei Patienten mit Autoimmunerkrankungen^28^ und auch bei Patienten mit SSc.^29^ Darüber hinaus zeigten Studien, dass NK‐Zellen bei SSc eine veränderte Zytokinproduktion und eine geringere zytotoxische Aktivität aufweisen.^30^, ^31^ Neben ihrer zytotoxischen Rolle besitzen NK‐Zellen auch antifibrotische Eigenschaften, wie die Unterdrückung der Leberfibrose.^32^ Ihre Verarmung könnte auch zu einer unkontrollierten Aktivierung von Immunzellen wie CD8^+^ T‐Zellen führen, die im Blut von Patienten mit Lungenbeteiligung erhöht waren. Dies deutet darauf hin, dass sich die Dysregulation des Immunsystems mit fortschreitender SSc von lokalisierten gewebespezifischen Reaktionen auf eine systemische Aktivierung verlagert, was erhebliche Auswirkungen auf Organe wie die Lunge hat.

Ein wichtiger Aspekt der Pathogenese von SSc ist das Vorhandensein von Autoantikörpern.^1^, ^5^ Bei Patienten, die Anti‐Scl‐70‐ oder CENP‐positiv waren, wurden unterschiedliche Immunsignaturen beobachtet. Patienten mit Anti‐Scl‐70‐Positivität zeigten eine Zunahme von CD8⁺ Effektor‐T‐Zellen in der Haut, was darauf hinweisen könnte, dass eine autoantikörpervermittelte Immunaktivierung zur fortschreitenden Gewebeschädigung bei der dcSSc beiträgt, bei der Fibrose und systemische Beteiligung ausgeprägter sind.^33^ Frühere Studien haben gezeigt, dass CD8^+^ T‐Zellen bei SSc‐Patienten hohe Mengen an profibrotischem IL‐13 produzieren, die Kollagensynthese fördern und eine Schlüsselrolle bei der Hautfibrose und Entzündung im Frühstadium spielen.^34^, ^35^ Umgekehrt ist bei CENP‐positiven Patienten, die in der Regel ein lcSSc aufweisen,^33^ ein Anstieg der ThGM‐CSF‐Zellen in der Haut zu beobachten, was ein weiterer Hinweis darauf ist, dass diese Zellen eine Schlüsselrolle beim milderen Verlauf der SSc spielen. Dieser Unterschied in den Immunprofilen zwischen Anti‐Scl‐70‐ und CENP‐positiven Patienten bestärkt die Idee, dass Autoantikörper die Immunantwort bei SSc steuern können, indem sie das klinische Bild prägen und die Immunzelllandschaft in verschiedenen Gewebekompartimenten beeinflussen. Somit unterstreicht diese Studie, wie wichtig es ist, den Autoantikörperstatus in Verbindung mit der Erstellung von Immunzellprofilen zu betrachten, um die Pathogenese und den Verlauf der SSc besser zu verstehen.

Zwar sind weitere Studien erforderlich, um diese Ergebnisse in einer größeren Patientenkohorte zu validieren, doch liefert diese Studie eine detaillierte Momentaufnahme der Immunlandschaft bei SSc und zeigt signifikante Veränderungen in Untergruppen von Immunzellen in verschiedenen Geweben, einschließlich der Haut, des subkutanen Fettgewebes und des Blutes. Diese Veränderungen sind mit wichtigen klinischen Parametern wie Hautfibrose, Lungenbeteiligung und Autoantikörperstatus verknüpft und verdeutlichen das komplexe Zusammenspiel zwischen dem angeborenen und adaptiven Immunsystem bei der Steuerung des Krankheitsverlaufs.

Es gibt mehrere Einschränkungen dieser Studie. Eine wesentliche Einschränkung ist die geringe Stichprobengröße der SSc‐Kohorte, die eine Validierung unserer Ergebnisse in einer größeren Patientenkohorte rechtfertigt. Außerdem können wir aufgrund des Studiendesigns und des Verzichts auf *counting beads* nur relative Werte der Zellsubtypen darstellen. Auch die unzureichende Übereinstimmung von Geschlecht und Alter zwischen Patienten und Kontrollen, das Fehlen von Analysen für Monozyten‐Untergruppen sowie das begrenzte Wissen über den Einfluss von Medikamenten auf Veränderungen der Immunzellprofile schränken die Schlussfolgerungen unserer Studie ein.

## DANKSAGUNG

Wir danken der *Core Facility* für Durchflusszytometrie der MedUni Wien für die Bereitstellung des Spektralanalysators Aurora (Cytek^®^). Diese Arbeit wurde durch eine Forschungsförderung der Medizinischen Gesellschaft für Oberösterreich (an MGS), den Österreichischer Wissenschaftsfonds (Grant‐DOI: 10.55776/PAT8019123 an GS) und die LEO Foundation (LF‐OC‐24‐001518 an GS) finanziell unterstützt. Außerdem wurde diese Arbeit vom Bundesministerium für Wirtschaft, Energie und Tourismus sowie von der Nationalstiftung für Forschung, Technologie und Entwicklung Österreichs im Rahmen des Christian Doppler Labors für chronisch‐entzündliche Hautkrankheiten unterstützt.

Open access Förderung durch die Medizinische Universität Wien/KEMO.

## FINANZIERUNG

Forschungsförderung der Medizinischen Gesellschaft für Oberösterreich (MGS), Österreichischer Wissenschaftsfonds (Grant‐DOI: 10.55776/PAT8019123) und LEO Foundation (LF‐OC‐24‐001518) (beides GS).

## INTERESSENKONFLIKT

Keiner.

## Supporting information



Supplementary information

Supplementary information

Supplementary information
