# Supplementary figures and images for: Zusammensetzung der Immunzellen in der Haut und dem subkutanen Fettgewebe von Patienten mit systemischer Sklerose
Source: J Dtsch Dermatol Ges. 2026 Apr 8;24(4):482–93. [Article in German] doi: 10.1111/ddg.15864_g (PMC13059057; doi:10.1111/ddg.15864_g)

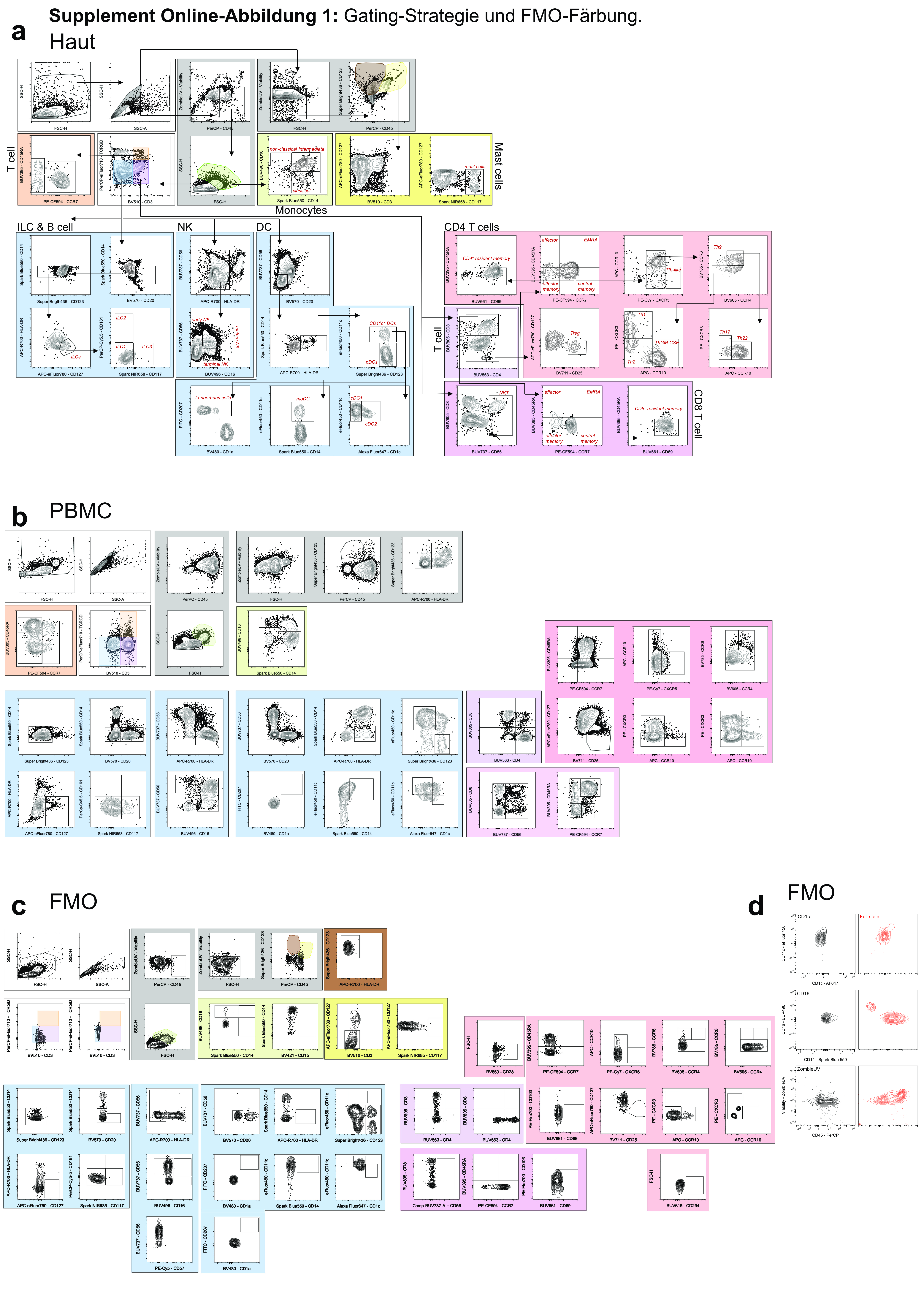

Supplement: Supplementary file 1 — Supplementary information [file DDG-24-482-s003.jpg]

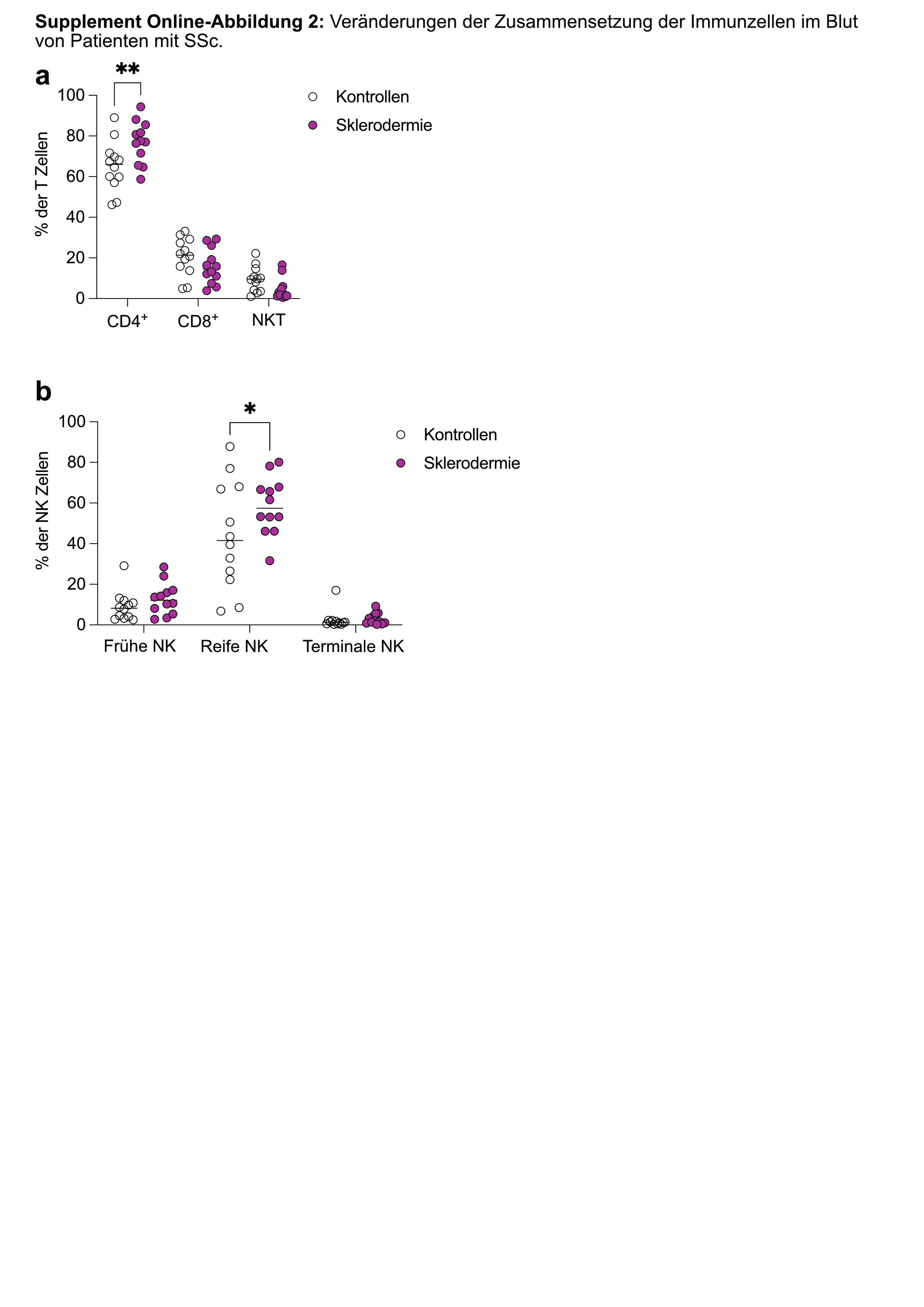

Supplement: Supplementary file 2 — Supplementary information [file DDG-24-482-s002.jpg]
