# Supplementary material for: Zusammensetzung der Immunzellen in der Haut und dem subkutanen Fettgewebe von Patienten mit systemischer Sklerose
Source: J Dtsch Dermatol Ges. 2026 Apr 8;24(4):482–93. [Article in German] doi: 10.1111/ddg.15864_g (PMC13059057; doi:10.1111/ddg.15864_g)
Supplement: Supplementary file 3 — Supplementary information [file DDG-24-482-s001.docx]

Table S1

| **Marker** | **Fluor** | **Clone** | **Supplier** | **Cat#** | **Dilution 1:X**  **(blood/tissue)** | **Laser** |
| --- | --- | --- | --- | --- | --- | --- |
| CD45RA | BUV395 | 5H9 | BD | 740315 | 400 / 400 | UV2 |
| Viability | ZombieUV | N/A | Biolegend | 423107 | 1000 / 1000 | UV6 |
| CD16 | BUC496 | 3G8 | BD | 612944 | 400 / 400 | UV7 |
| CD4 | BUV563 | OKT4 | BD | 750979 | 400 / 200 | UV9 |
| CD294 | BUV615 | BM16 | BD | 751216 | 200 / 400 | UV10 |
| CD69 | BUV661 | FN50 | BD | 750213 | 100 / 400 | UV11 |
| CD56 | BUV737 | NCAM 16.2 | BD | 564447 | 200 / 50 | UV14 |
| CD8 | BUV805 | SK1 | BD | 612889 | 200 / 400 | UV16 |
| CD15 | BV421 | W6D3 | BD | 740086 | 200 / 200 | V1 |
| CD123 | SuperBright 436 | 6H6 | ThermoFisher | 62-1239-42 | 100 / 200 | V2 |
| CD11c | eFluor 450 | 3.9 | ThermoFisher | 48-0116-42 | 200 / 200 | V3 |
| CD1a | BV480 | HI194 | BD | 566147 | 100 / 100 | V5 |
| CD3 | BV510 | SK7 | Biolegend | 344828 | 400 / 200 | V7 |
| CD20 | BV570 | 2H7 | Biolegend | 302332 | 200 / 100 | V8 |
| CCR4 | BV605 | L291H4 | Biolegend | 359418 | 50 / 100 | V10 |
| CD28 | BV650 | CD28.2 | Biolegend | 302946 | 100 / 100 | V11 |
| CD25 | BV711 | 2A3 | BD | 563159 | 100 / 100 | V13 |
| CCR6 | BV785 | G034 | Biolegend | 353422 | 50 / 20 | V15 |
| CD207 | FITC | MB22-9F5 | Miltenyi Biotec | 130-098-349 | 100 / 100 | B2 |
| CD14 | SparkBlue 550 | 63D3 | Biolegend | 367148 | 200 / 100 | B3 |
| CD45 | PerCP | HI30 | Biolegend | 304026 | 400 / 200 | B8 |
| CD161 | PerCP-Cy5.5 | HP-3G10 | Biolegend | 339908 | 200 / 400 | B9 |
| TCRGD | PerCP-eFluor 710 | B1.1 | ThermoFisher | 46-9959-42 | 200 / 400 | B10 |
| CXCR3 | PE | 1C6 | BD | 557185 | 50 / 50 | YG1 |
| CCR7 | PE-CF594 | 150503 | BD | 562381 | 100 / 100 | YG3 |
| CD57 | PE-Cy5 | NK-1 | SouthernBiotech | 9665-13 | 100 / 50 | YG5 |
| CD103 | PE/Fire700 | Ber-ACT8 | Biolegend | 350240 | 100 / 200 | YG7 |
| CXCR5 | PE-Cy7 | J252D4 | Biolegend | 356924 | 200 / 200 | YG9 |
| CCR10 | APC | 314305 | R&D | FAB3478A-100 | 50 / 100 | R1 |
| CD1c | Alexa Fluor 647 | L161 | Biolegend | 331510 | 100 / 200 | R2 |
| CD117 | SparkNIR 685 | 104D2 | Biolegend | 313250 | 200 / 200 | R4 |
| HLA-DR | APC-R700 | G46-6 | BD | 565127 | 200 / 200 | R5 |
| CD127 | APC-eFluor 780 | RDR5 | ThermoFisher | 47-1278-42 | 200 / 50 | R7 |

**Table S2:**

|  | **Skin** | **Fat** |
| --- | --- | --- |
| C1 | 1,117.6 | 238.9 |
| C2 | 1,638.6 | 113.1 |
| C3 | 371.3 | 134.6 |
| C4 | 3,939.2 | 116.0 |
| C5 | 49.7 | 42.0 |
| C6 | 815.1 | 235.0 |
| C7 | 318.6 | 149.6 |
| C8 | 1,433.7 | 299.2 |
| C9 | 1,392.3 | 211.3 |
| C10 | 1,398.8 | 25.3 |
| C11 | 5,956.8 | 272.6 |
| C12 | 1,245.4 | 123.1 |
| SSd lesional 1 | 712.3 | 127.2 |
| SSd lesional 2 | 3030.0 | 18.9 |
| SSd lesional 3 | 463.3 | 38.0 |
| SSd lesional 4 | 2,111.9 | 160.7 |
| SSd lesional 5 | 2,349.6 | 185.5 |
| SSd lesional 6 | 2,791.2 | 260.2 |
| SSd lesional 7 | 1,500.5 | 436.5 |
| SSd lesional 8 | 1,149.2 | 22.3 |
| SSd lesional 9 | 1,248.9 | 104.0 |
| SSd lesional 10 | 5,843.0 | 80.7 |
| SSd lesional 11 | 1,330.9 | 1131.6 |
| SSd lesional 12 | 622.4 | 297.3 |
| SSd lesional 13 | 2,656.6 | 107.8 |
| SSd non-lesional 1 | 5,063.4 | 187.0 |
| SSd non-lesional 2 | 1,030.2 | 493.9 |
| SSd non-lesional 3 | 521.5 | 84.5 |
| SSd non-lesional 4 | 703.9 | 482.5 |
| SSd non-lesional 5 | 3,023.0 | 149.2 |

*Abbr.:* C, control; SSd, systemic sclerosis
